# Cognitive impairment in an animal model of multiple sclerosis and its amelioration by glatiramer acetate

**DOI:** 10.1038/s41598-019-40713-4

**Published:** 2019-03-11

**Authors:** Rina Aharoni, Nofar Schottlender, Dekel D. Bar-Lev, Raya Eilam, Michael Sela, Michael Tsoory, Ruth Arnon

**Affiliations:** 1 Department of Immunology, The Weizmann Institute of Science, Rehovot, 761001 Israel; 2 Department of Veterinary Resources, The Weizmann Institute of Science, Rehovot, 761001 Israel

## Abstract

The severe motor impairment in the MS animal model experimental autoimmune encephalomyelitis (EAE) obstructs the assessment of cognitive functions. We developed an experimental system that evaluates memory faculties in EAE-affected mice, irrespective of their motor performance, enabling the assessment of cognitive impairments along the disease duration, the associated brain damage, and the consequences of glatiramer acetate (GA) treatment on these manifestations. The delayed-non-matching to sample (DNMS) T-maze task, testing working and long term memory was adapted and utilized. Following the appearance of clinical manifestations task performances of the EAE-untreated mice drastically declined. Cognitive impairments were associated with disease severity, as indicated by a significant correlation between the T-maze performance and the clinical symptoms in EAE-untreated mice. GA-treatment conserved cognitive functions, so that despite their exhibited mild motor impairments, the treated mice performed similarly to naïve controls. The cognitive deficit of EAE-mice coincided with inflammatory and neurodegenerative damage to the frontal cortex and the hippocampus; these damages were alleviated by GA-treatment. These combined findings indicate that in addition to motor impairment, EAE leads to substantial impairment of cognitive functions, starting at the early stages and increasing with disease aggravation. GA-treatment, conserves cognitive capacities and prevents its disease related deterioration.

## Introduction

Multiple sclerosis (MS) has traditionally been regarded as a demyelinating inflammatory disease that focally affects the central nervous system (CNS) white matter. However, improvements in histopathological analyses and advanced imaging have clearly revealed that MS is a global CNS disease, involving also the gray matter^1,2^. Furthermore, it is now recognized that gray matter damage in MS is widespread, underlying the long-term irreversible neurological impairment, especially the cognitive deterioration, manifested in up to 70% of patients as deficits in memory, learning, attention, processing speed or executive functions^3–7^. Despite the recognition in cognitive deficits as key symptoms of MS, experimental evidence regarding the ability of disease-modifying treatments to affect it is still sparse.

Cognitive impairment is detected in the earliest clinical stages^8^, yet progressive MS patients generally display more pronounced and pervasive deficits^5,9^. This is probably due to the extensive neurodegenerative pathological occurrence at disease progression. Indeed, cognitive deterioration strongly correlates with the demyelination, neuronal loss, cortical thinning and atrophy affecting the gray matter^1,9^. The cognitive domains affected during MS are mediated via largely distributed networks, including the prefrontal cortex, thalamus, basal ganglia, and hippocampus^2,6,10–13^, and many aspects of their associated pathologies still need to be investigated. Animal models can be useful to analyze specific cognitive deficits, their connection to tissue damage, and the ability to affect them by therapy. However, the severity of the motor impairments in the MS animal model - experimental autoimmune encephalomyelitis (EAE) impedes its utilization for such studies. Consequently, cognitive impairments in EAE-affected animals were documented either during the pre-symptomatic stage, or early in the disease course^14^. In other experiments, animals with advanced clinical scores were excluded, so cognitive parameters were measured only in animals with low clinical scores^15,16^.

The therapeutic effect of Glatiramer acetate (GA, Copaxone), a disease-modifying therapy (DMT) for MS, is mainly attributed to immunomodulation, inducing deviation from the pro-inflammatory to the anti-inflammatory pathways^17,18^. Accumulated findings indicate that GA-treatment of EAE-affected mice leads also to augmentation of neuroprotective processes, such as elevation in neurotrophic factors including brain derived neurotrophic factor (BDNF)^19,20^. Utilizing immunohistochemistry and electron microscopy in different EAE models, we found protective outcome of GA on the primary disease target, the myelin^21,22^. Ultrastructural quantitative analysis provided evidence also for significant increase in remyelination after GA-treatment^22,23^. The mode of action of GA in this system is attributed to an increase in proliferation of oligodendrocyte progenitor cells and their recruitment into injury sites^21^. Furthermore, GA-treatment augmented neuronal progenitor cells proliferation to a higher level than that observed in EAE-untreated mice, and this effect persisted for a prolonged duration^24^. Neuronal progenitors were seen diverging from the classic migratory streams and spreading to damage sites in brain regions that do not normally undergo neurogenesis. Findings from human studies support the notion that GA confers neuroprotection in MS patients. GA treatment reduced the formation of permanent T1 hypo-intense lesions that evolve into “black holes”, which have been associated with irreversible neurological disability^25^. Using quantitative MRI analysis, it was also shown that GA-treatment of MS patients for one year leads to a significant increase in the NAA:Cr ratio compared to pre-treatment values, implying axonal metabolic recovery and protection from sub-lethal axonal injury^26^. These neuroprotective and repair processes may counteract the neurodegenerative disease-pathology that underlie cognitive impairments.

We have recently demonstrated that under the inflammatory conditions in the cortex of EAE-mice, activated perivascular astrocytes detach from the blood vessels and fail their role in neuro-hemodynamic coupling, resulting in obstructed crosstalk between the blood vessels and the neurons^27^. Immunomodulatory GA-treatment, either before or after clinical symptom manifestations, abrogated this neurovascular aberration. We proposed that loss of cortical astrocytic regulation and fine-tuning between the blood supply and the neuronal needs can lead to neurological impairments and cognitive decline. However, cognitive dysfunctions in EAE-mice during the advance stages of the disease, as well as the ability to prevent such impairment by GA-treatment, have not yet been determined.

In view of the above findings, we aimed to devise a new experimental approach to behaviorally evaluate cognitive functions in EAE-affected mice, irrespective of motor impairments, thus applicable even at the peak of the disease. The traditional delayed-non-matching to sample (DNMS) T-maze was adapted to minimize the physical demands and to focus on the choices the mice made, revealing working and long-term memory deficits. This enabled specific assessment of cognitive deficits along the disease duration and evaluation of the associated tissue damage. We report herewith that the EAE disease process leads to substantial working and long-term memory impairments. GA treatment conserved cognitive functions, so that despite their exhibited mild motor impairments, the treated mice performed similar to naïve controls. Furthermore, we show that the EAE-induced cognitive deficits coincide with gray matter damages in the frontal cortex and throughout the hippocampus, and these damages can be reduced by GA-treatment.

## Results

**Figure 1:**
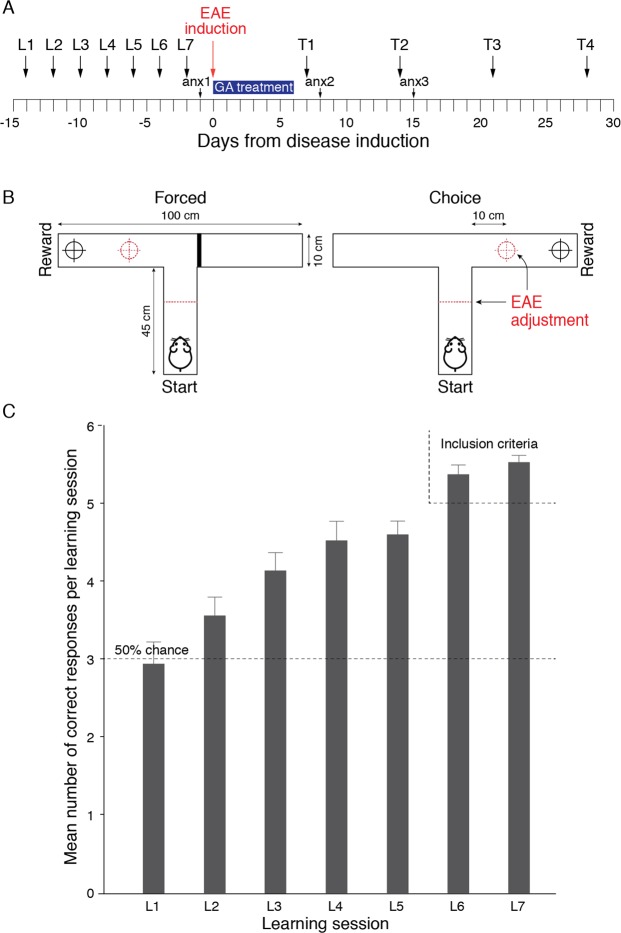
Experimental layout and the DNMS T-maze Task. (**A**) Experimental time-line. L - learning session; T - Testing session, anx - anxiety-like behavioral test. (**B**) A schematic depiction of the T-maze apparatus and its adjustment to EAE-affected mice (dashed lines). An illustrated example of one trial comprised of a ‘forced’ run in which the reward is located in the left arm, and a ‘choice’ run in which the reward is located in the right arm. (**C**) Acquiring the DNMS T-maze task. Training was performed every other day for seven consecutive learning sessions (L1–L7), each daily session consisting of a set of six forced-choice runs. The number of correct responses (Mean ± SEM) in the ‘Choice’ runs, of 35 mice that achieved the inclusion criterion (at least five correct responses for both L6 and L7) is shown.

To evaluate the cognitive deficits inflicted by EAE, when motor impairments impede tasks performance, we designed an experimental scheme that allowed assessing long term and working memory faculties, irrespective of motor functions. The traditional delayed-non-matching to sample (DNMS) T-maze that assess working memory, was adapted and utilized. The experimental outline of the study is depicted in Fig. 1A.

### Acquiring the DNMS T-maze task

Prior to disease induction, all the mice were taught a “rule” that the reward food, in the ‘choice’ runs, is always located at the opposite arm from the arm in which it was placed in the previous ‘forced’ runs (either left or right, randomly interchangeable between trials, example illustrated in Fig. 1B). Training was performed in seven sessions (learning sessions L1–L7, every other day, days −15 to −2 in Fig. 1A), each daily session consisted of six ‘forced-choice’ runs. Accordingly, to learn the rule, in each ‘choice’ run the mice had to rely on their working memory to remember where they were forced to turn in the previous ‘forced’ run. Whereas, to apply the rule over the daily sessions, the mice had to rely on long term memory functions. An inclusion criterion to take part in the experiment was at least five correct responses at both L6 and L7. The learning curve of the mice that achieved the inclusion criterion (35/50 mice) is shown in Fig. 1C. As seen, the initial mice performance was similar to that expected by chance. Thereafter, the number of correct responses gradually increased, to a mean of 5.50 ± 0.11 (out of six) at the 7^th^ training session, indicating that the mice have successfully acquired the task.

Friedman’s two-way ANOVA by ranks indicated a significant increase over training [χ^2^_(6)_ = 70.126; p < 0.001; mean ranks- L1 = 2.57, L2 = 2.67, L3 = 3.66, L4 = 4.09, L5 = 4.11, L6 = 5.34, L7 = 5.56]. Wilcoxon signed-rank tests (Bonferroni corrected) pairwise comparisons indicated that performance at L7 was significantly better than at L5 (p = 0.030), L4 (p = 0.024) and L1-3 (p < 0.001).

### The effect of EAE induction and GA treatment on motor dysfunction

**Figure 2:**
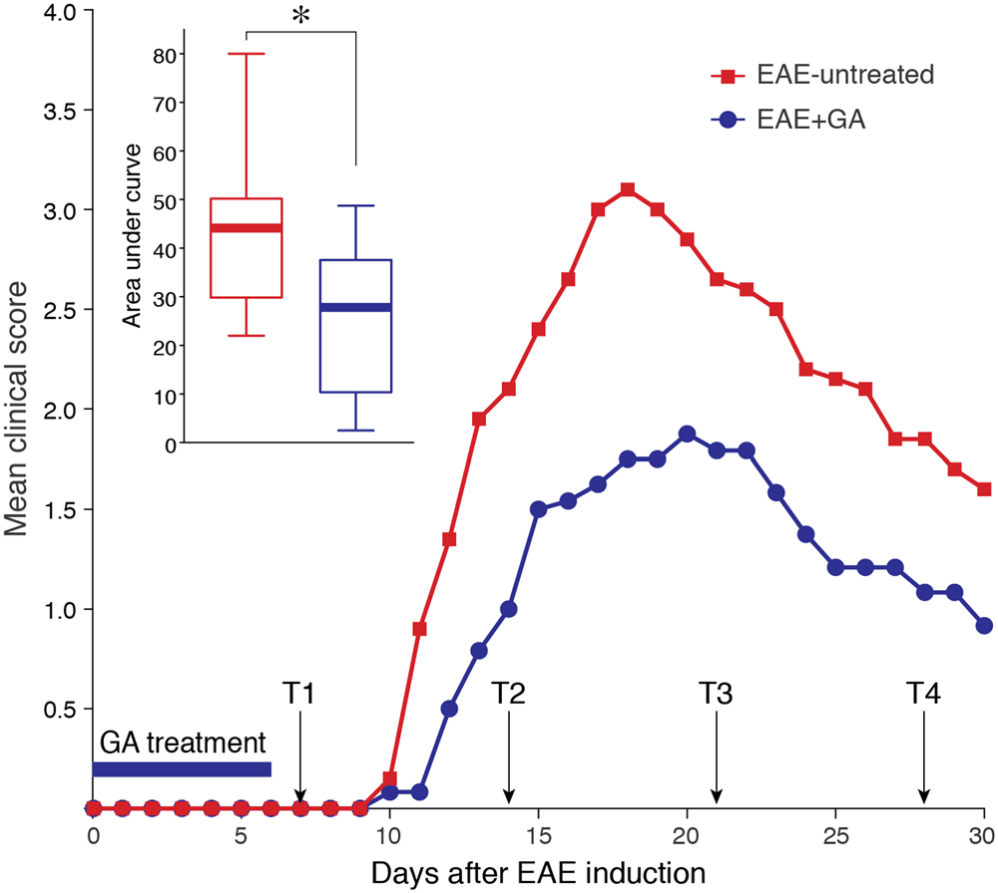
The effect of EAE induction and GA treatment on motor dysfunction. The daily mean clinical scores of mice induced by the MOG 35–55 peptide EAE model, 12 PBS-injected (EAE-untreated), and 13 GA-injected (EAE + GA) 2 mg/mouse daily, starting at the day of disease induction. EAE was scored as follows: 1 - loss of tail tonicity, 2 - hind limb weakness or partial paralysis, 3 – hind leg paralysis, 3.5 hind leg complete paralysis with hind body paresis, 4 - hind and foreleg paralysis, 5 - death. Insert depicts the area under curve for days 0–30. Data distribution for each group is presented by box plots, in which the center lines show the medians, box limits indicate the 25^th^ and 75^th^ percentiles, whiskers indicate the minimal and maximal values. *Indicates significant changes (p = 0.015).

The mice that achieved the inclusion criterion were divided into three groups with similar mean correct responses at L6 and L7, naïve (n = 10), EAE-induced mice injected with PBS alone (EAE-untreated, n = 12), and EAE-induced mice treated with GA (EAE + GA, n = 13). Disease was induced one day after the last learning session, using the MOG 35–55 peptide-induced chronic EAE model. The daily mean clinical scores (determined according to the manifested motor impairments, Fig. 2) indicate that in EAE-untreated mice, motor impairments typically appeared 10–12 days after disease induction, increasing in severity and reaching a mean score of 3.10 ± 0.98 and 3.00 ± 0.95 (complete hind body paralysis) by days 17–18, respectively. Thereafter, motor impairments gradually decreased to a mean score of 1.60 ± 0.51 one month after disease induction. Mice treated with GA (seven consecutive daily injections, starting at the day of disease induction) exhibited milder motor dysfunctions, reaching a maximal mean score of 1.79 ± 0.52 on day 20, and 0.92 ± 0.26 one month after disease induction. A significant difference [t_(20)_ = 2.672; p = 0.015] was evident for the area-under-curve parameter (AUC, insert in Fig. 2), which indicates the combined clinical scores for the entire experimental period (days 0–30); EAE untreated: 43.85 ± 5.22, EAE + GA: 25.29 ± 4.61.

### The effect of EAE induction and GA treatment on performance in the DNMS T-maze task

To test cognitive functions in EAE-affected mice, when the motor impairment impedes their mobility, we adjusted the T-maze apparatus by relocating the start and reward positions closer to the T junction of the maze (10 cm instead of 45 cm, Fig. 1B). In addition, for mice with clinical grades of two and above, no time limitation to complete a run was imposed. Thus, the T-maze score was solely based on the choices made by the mice, and did not take into account indices affected by their motor impairments.

**Figure 3:**
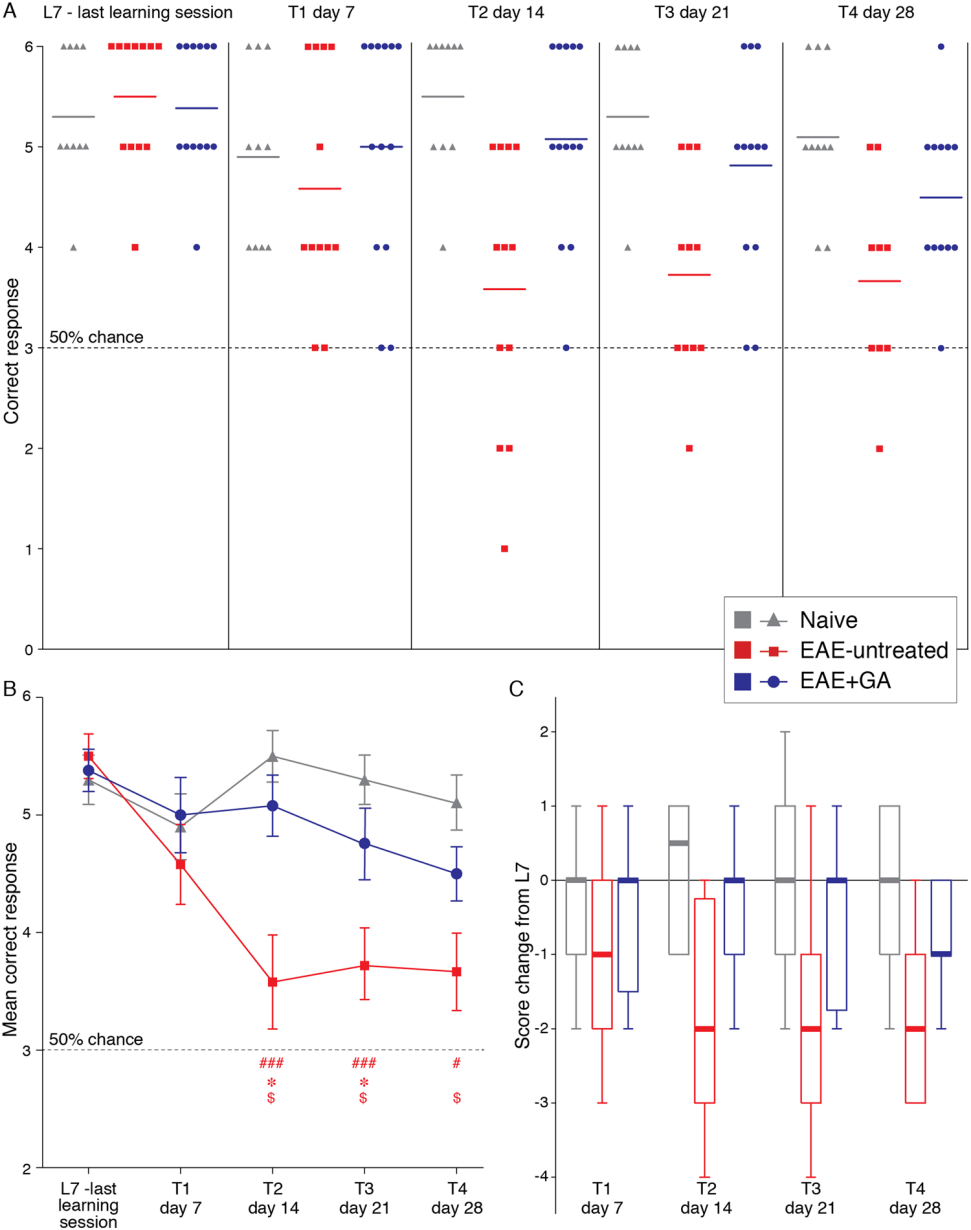
The effects of EAE induction and GA-treatment on DNMS T-maze task performance. The correct responses (out of six forced-choice runs per session) of 10 naive, 12 EAE-untreated, and 13 EAE + GA mice, performed at the last learning session (L7), and at days 7 (T1), 14 (T2), 21 (T3), 28 (T4) after disease induction. (**A**) The detailed numbers of correct responses of individual mice. (**B**) The mean correct response per group ± SEM. (**C**) Score changes from the score at the last training session (L7) presented by box plots, in which the center lines show the medians, box limits indicate the 25^th^ and 75^th^ percentiles, whiskers indicate the minimal and maximal values. ^#^Indicates significant differences of EAE-untreated mice from naïve mice (^#^p < 0.05, ^##^p < 0.01, ^###^p < 0.005), *Indicates significant differences of EAE-untreated mice from EAE + GA mice (p < 0.05), ^$^indicates significant changes in the performance of EAE-untreated mice from their performance at L7 (p < 0.05).

The combined DNMS T-maze choices of 10 naive, 12 EAE-untreated, and 13 EAE + GA mice, performed 7 (T1), 14 (T2), 21 (T3) and 28 (T4) days after disease induction, are presented in Fig. 3, as the detailed numbers of correct responses in the ‘choice’ runs (out of six forced-choice runs per session) of individual mice (A), the mean correct response per group (B), and the score changes from the end of learning (L7) (C). At L7, groups’ performances were indistinguishable (naïve 5.30 ± 0.21, EAE-untreated 5.50 ± 0.19, and EAE + GA 5.38 ± 0.18). However, over the experimental period, task performance differentially changed across the groups. Among EAE-untreated mice, task performance drastically declined, particularly after manifestation of clinical symptoms, at days 14, 21, and 28, (mean correct responses 3.58 ± 0.43, 3.72 ± 0.33, and 3.66 ± 0.40, respectively), approaching the performance of the early training sessions (median score change 2 from L7 for all three time points). In contrast, GA-treatment attenuated the decline in performance (mean correct responses 5.00 ± 0.32, 5.08 ± 0.26, 4.67 ± 0.33, 4.50 ± 0.23, on days 7, 14, 21 and 28 from disease induction, respectively, no change in the median score from L7 at days 14 and 21).

Friedman’s two-way ANOVA by ranks, indicated a statistically significant deterioration in performance throughout the course of the experiment only in the EAE-untreated group [χ^2^_(4)_ = 13.707; p = 0.008; mean ranks- L7 = 4.39, T1 = 3.28, T2 = 2.17, T3 = 2.83, T4 = 2.33]. Wilcoxon signed-rank tests (Bonferroni corrected) pairwise comparisons indicated that compared with L7, untreated EAE induced mice performed significantly worse at T2 (p = 0.028), T3 (p = 0.024), and T4 (p = 0.044). The performance of the naive and GA-treated mice did not change throughout the experiment [naïve: χ^2^_(4)_ = 2.726; p = 0.605. GA-treatment: χ^2^_(4)_ = 7.506; p = 0.111].

Additional Kruskal-Wallis analysis sought for differences in the number of correct responses between the groups within each time point, revealed significant differences between the groups at T2, T3 and T4 [T2: χ^2^_(2)_ = 13.364; p = 0.001; mean ranks- naive = 24.50, EAE-untreated = 9.88, EAE + GA = 20.50. T3: χ^2^_(2)_ = 11.901;p = 0.003; mean ranks- naive = 22.70, EAE-untreated = 9.45, EAE + GA = 19.17. T4: χ^2^_(2)_ = 7.538;p = 0.023; mean ranks- naive = 21.35, EAE-untreated = 10.50, EAE + GA = 15.67]. Dunn’s (Bonferroni corrected) pairwise comparisons indicated that EAE-untreated mice performed significantly worse than both naive and GA-treated mice at T2 and T3 (T2: EAE < naive p = 0.002; EAE-untreated <EAE + GA p = 0.021. T3: EAE-untreated <naive p = 0.003; EAE-untreated <EAE + GA p = 0.034). At T4, the EAE-untreated mice performed significantly worse than naive mice (EAE < naive p = 0.018). The groups did not differ at any other time point [L7: χ^2^_(2)_ = 0.635; p = 0.728; T1: χ^2^_(2)_ = 0.977; p = 0.607].

Throughout the experiment, GA-treated mice performance did not differ from that of naïve controls, although they reached a clinical score of nearly 2 (at day 21). Additional Wilcoxon signed-rank test further indicated that GA-treated mice performance at day 21 did not differ from their performance at L7 [Mdn = 5.000 for both, Z = 1.518; p = 0.129].

**Figure 4:**
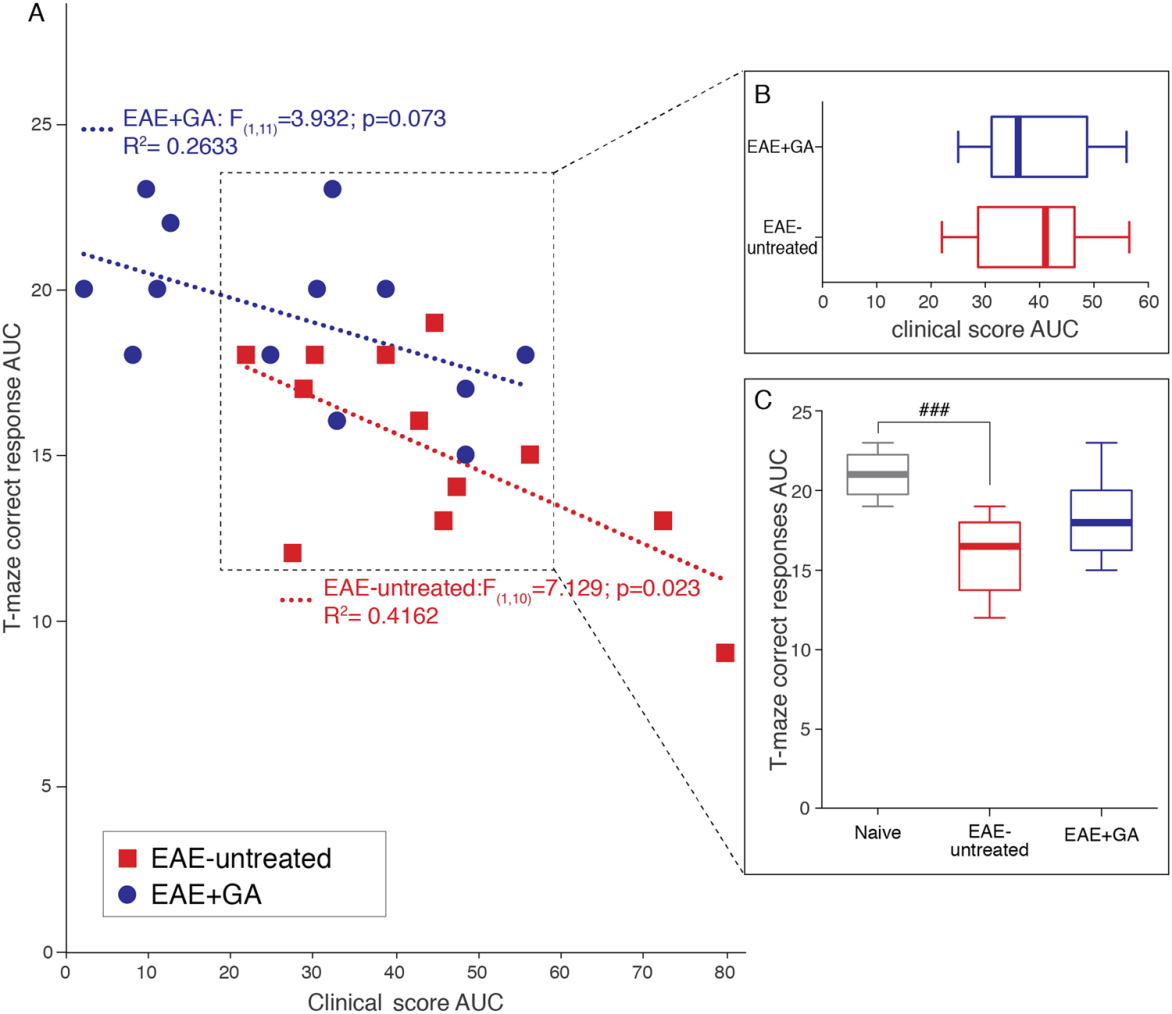
The relationship between T-maze task performance and motor dysfunction. (**A**) A scatter-plot depicts the relationship between the overall DNMS T-maze task performance (correct responses T1-T4 AUC) and motor symptoms presented throughout the experimental period (clinical score AUC) for the EAE-untreated group and the EAE + GA-treated group. A simple linear regression equation that predicts correct responses T1-T4 AUC based on clinical score AUC is depicted for each group. A significant (p < 0.05) regression (R^2^ = 0.416) was found for the EAE-untreated, but not for the EAE + GA-treated group. (**B**,**C**) Additional analyses of EAE untreated (n = 10) and EAE GA-treated (n = 8) sub-populations (dashed box) that presented similar overall motor impairments (**B**). Only the EAE untreated mice exhibited significat impaired T-maze performance comapred to naïve mice (n = 10) (**C**). Data presented by box plots, in which the center lines show the medians, box limits indicate the 25^th^ and 75^th^ percentiles, whiskers indicate the minimal and maximal values. AUC - area-under-curve, ^###^Indicates significant difference of EAE-untreated mice compared to naïve mice (p < 0.001).

In order to understand whether EAE-untreated mice performed worse than others only because they exhibited more pronounced clinical manifestations, the relationship between cognitive and motor impairments were delineated. First the relationship between overall task correct responses T1–T4 area under the curve (T-maze AUC) and overall motor symptoms (clinical score AUC) was assessed by calculating a linear regression for the EAE-untreated and the EAE + GA-treated groups (Fig. 4A). A significant regression equation was found for the EAE-untreated group [F_(1,10)_ = 7.129; p = 0.023], with an R^2^ of 0.416, but not for the EAE + GA-treated group [F_(1,11)_ = 3.932; p = 0.073], indicating that task performance of EAE-untreated mice was strongly associated with disease severity, whereas in GA-treated mice task performance was unrelated to the motor disease burden.

Further analyses assessed the overall task performance of mice presenting similar motor impairments. T-maze AUC of EAE-untreated and EAE + GA-treated mice were compared while controling for overall motor symptoms. These analyses included only EAE-induced mice with a clinical score AUC greater than 20 and smaller than 60 (Fig. 4A dashed box), comprising 10 EAE-untreated and 8 EAE + GA-treated mice. These sub-populations were indistiguishable in their clinical score AUC [t_(16)_ = 0.116; p = 0.909], (Fig. 4B). However, as illustrated in Fig. 4C, one way ANOVA indicated a significant difference between these sub-populations and naïve mice (n = 10) [F_(2, 27)_ = 12.690; p < 0.001]. Scheffe post-hoc comparisons indicated that only EAE-untreated mice performed significantly worse than naïve mice (p < 0.001). All other post-hoc comparisons were not significant [naïve~EAE + GA (p = 0.075); EAE-untreated~EAE + GA (p = 0.082)]. These analyses imply that the differences in T-maze task performance between EAE-untreated and EAE + GA mice cannot be attributed to their differences in motor functions.

Collectively, these analyses indicate that in addition to the motor impairments, the EAE disease process impairs cognitive functions. GA treatment conserved cognitive capacities, so that despite their exhibited (mild) motor impairment, the treated mice performed similar to naïve controls.

Since anxiety is known to alter cognitive functions^28,29^ anxiety-like behaviors were assessed in parallel to the T-maze assessments. The detailed findings obtained at the anxiety tests are described in the supplementary information and in Supplementary Fig. 1. Collectively, the anxiety indices data indicate that the differences in the T-maze task performance found between EAE-mice, untreated as opposed to GA treated, are not related to differences in anxiety.

### The effect of EAE induction and GA treatment on frontal cortex and hippocampus brain damages

**Figure 5:**
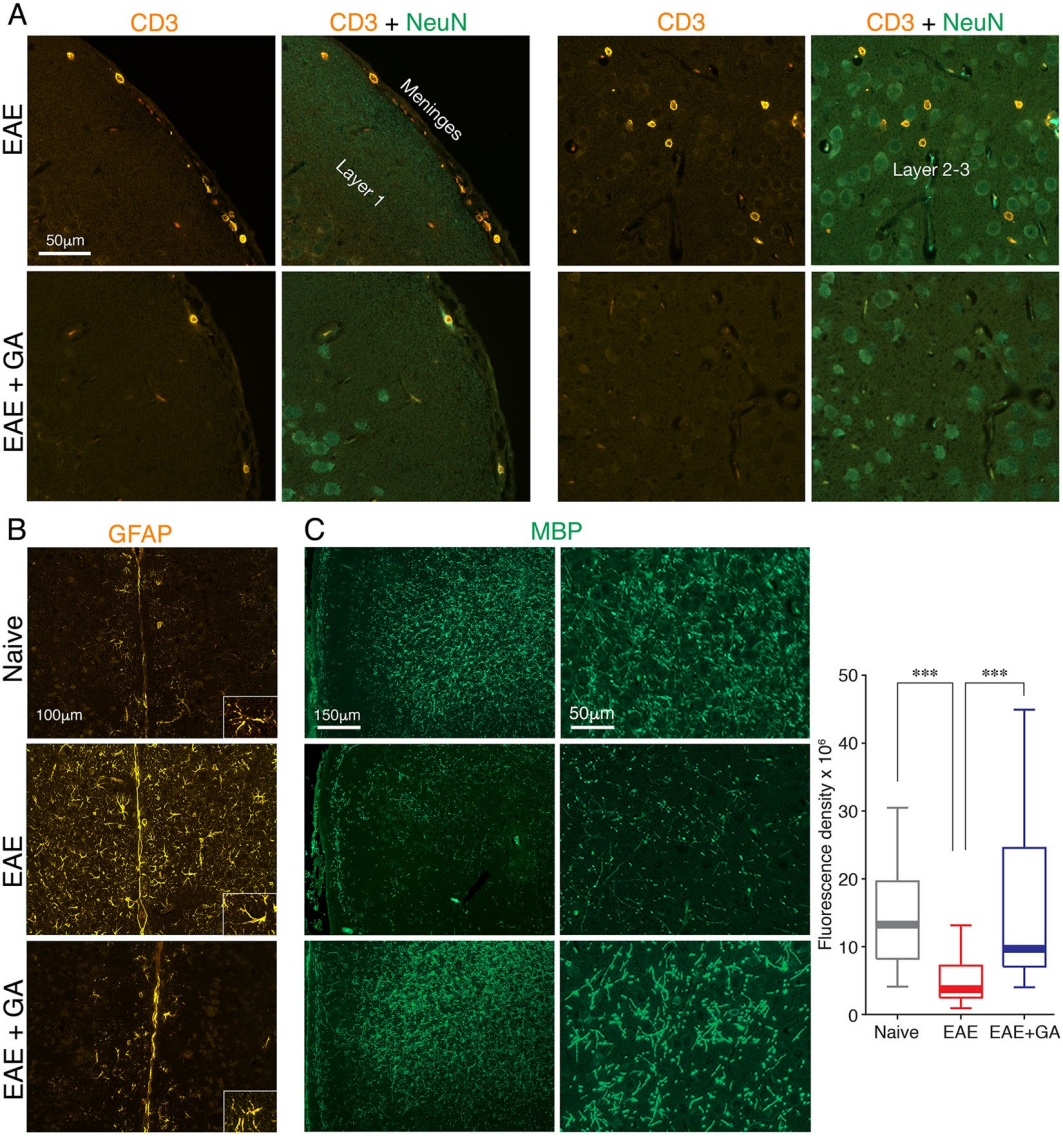
The effect of EAE induction and GA-treatment on brain damages in the frontal cortex. Immunohistochemical analyses were performed in brains of EAE-induced untreated mice (EAE), EAE-induced mice treated by GA (EAE + GA), and naïve controls, 21 days after disease induction. (**A**) Staining for CD3 expressing T-cells and NeuN expressing neurons. Left: T-cells in the meninges (shown in the pial upper surface). Right: T-cells that penetrated into the frontal cortex (shown in layers 2–3). (**B**) Staining for GFAP expressing astrocytes, revealing in EAE-untreated-mice widespread astrogliosis (shown in layer 1 adjacent to the midline). (**C**) Staining for myelin by MBP-antibodies, revealing extensive demyelination in EAE-untreated-mice. Depictions from two different mice from each group, and quantification of MBP expression are shown. Fluorescence density of MBP expression was measured in cortical layers I–III, in areas of 27,900 μm^2^, 6–8 measurements from both hemispheres per mouse, 4 mice per group. Data presented by box plots, in which the center lines show the medians, box limits show the 25^th^ and 75^th^ percentiles, whiskers indicate the minimal and maximal values. ***Indicates significant differences (p < 0.005).

**Figure 6:**
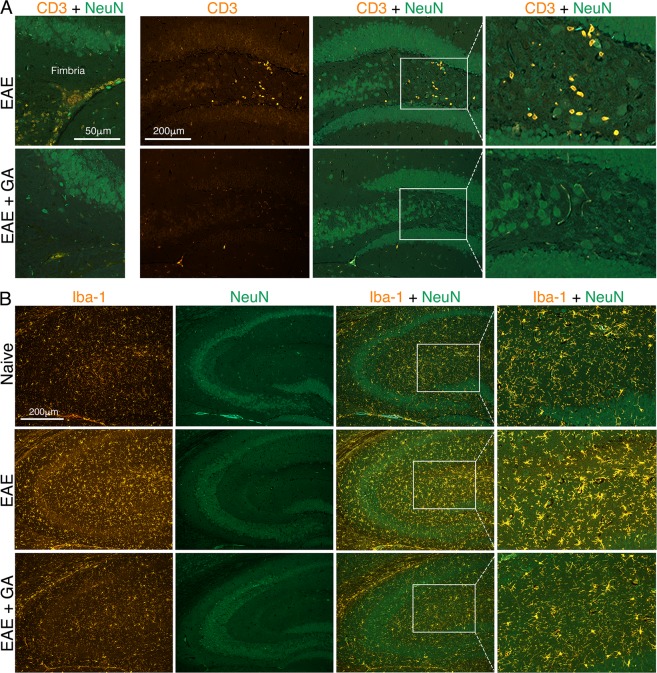
The effect of EAE induction and GA-treatment on hippocampus inflammation. Immunohistochemical analyses were performed in brains of EAE-induced untreated mice (EAE), EAE-induced mice treated by GA (EAE + GA), and naïve controls, 21 days after disease induction. (**A**) Staining for CD3 expressing T-cells and NeuN expressing neurons. Left: T-cells in the fimbria hippocampus. Right: T-cells in the hippocampus dentate gyrus. (**B**) Staining for Iba-1 expressing microglia astrocytes, revealing in EAE-untreated-mice extensive microglia activation. Note the dotted lines, indicating that the right column in A and B depict high magnification images of the picture in the inset.

**Figure 7:**
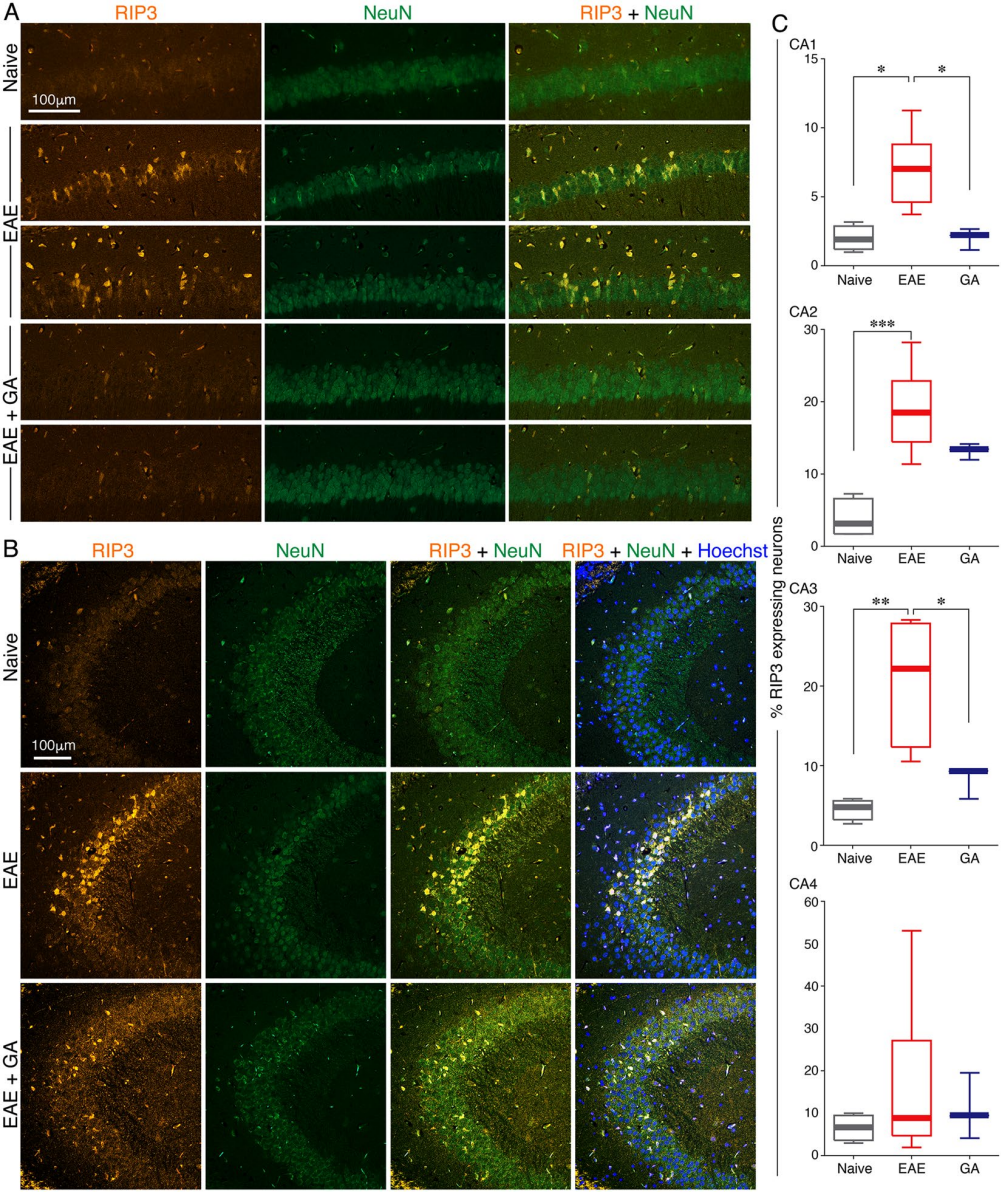
The effect of EAE induction and GA-treatment on hippocampus neuronal injury. Immunohistochemical analyses were performed in brains of EAE-induced untreated mice (EAE), EAE-induced mice treated by GA (EAE + GA), and naïve controls, 21 days after disease induction. Staining for necroptotic (RIP3 expressing) neurons (NeuN^+^) (**A**) in the CA1 and (**B**) in the CA3 segments of the hippocampus. (**C**) Quantitative analysis of necroptotic neurons performed by counting the number of RIP3 positive cells from the total number of NeuN positive neurons in CA1 (47,850 μm^2^ per section), CA2 (12,960 μm^2^ per section), CA3 (59,400 μm^2^ per section), and CA4 (11,250 μm^2^ per section), 6–8 quantifications from both hemispheres per mouse, 3–7 mice per group. Data presented by box plots, in which the center lines show the medians, box limits indicate the 25^th^ and 75^th^ percentiles, whiskers indicate the minimal and maximal values. *Indicates significant differences (*p < 0.05, **p < 0.01).

To assess whether the poor performance in the T-maze relates to disease induced tissue damage, we looked for the disease hallmarks: inflammation, demyelination, and neuronal death, in the frontal regions of the cortex (Fig. 5) and in the hippocampus (Figs 6 and 7), which have been associated with this task performance^30–33^. Immunohistochemical analyses were performed in brains of EAE-induced untreated mice (EAE), and EAE-induced mice treated by GA (EAE + GA), compared to naïve controls (3–7 mice per group), 21 days after disease induction, corresponding to time-point T3, in which a significant decline in T-maze performance was found in the EAE, but not in the EAE + GA mice.

Staining by anti-CD3 antibodies revealed in EAE-untreated mice, cell accumulations containing CD3 expressing T-cells in the meninges (shown in the pia on the upper cortical surface in Fig. 5A, left), as well as in white matter regions (shown in the fimbria hippocampus fibers Fig. 6A, left). T-cells that penetrated into the frontal cortex (shown in layers 2–3 in Fig. 5, right) and into the hippocampus (shown in the dentate gyrus in Fig. 6, right) were also detected in EAE-mice. In addition, we detected in untreated mice, in these regions, widespread astrogliosis, namely, astrocytes with increased GFAP expression, retracted un-branched processes, and enlarged cell bodies (demonstrated in the frontal cortex in Fig. 5B). Iba-1 positive microglia also manifested characteristic activated morphology, retraction and thickening of their processes and enlarged cell soma (demonstrated in the hippocampus in Fig. 6B). In contrast, in EAE + GA mice, cellular infiltrations were typically smaller and less frequent. Furthermore, astrocyte and microglia activation was either moderate or absent and their morphology resembled the typical pre-inflammatory appearance. Thus, T-cell infiltration and reactive gliosis, the hallmarks of CNS inflammation, which were prevalent in the frontal cortex and the hippocampus of EAE-affected mice, were down-regulated following GA-treatment. In the cortex of naïve mice, we did not detect CD3^+^ T-cells or astrocyte/microglia activation.

Myelin damage, assessed using MBP antibodies, revealed extensive demyelination in the frontal cortex of EAE-untreated mice (depictions of two different mice from each group shown in Fig. 5C), 71% reduction in the median fluorescence density of MBP expression from naïve mice (measured in cortical layers I–III, in areas of 27,900 μm^2^, 6–8 measurements from both hemispheres per mouse, four mice per group). Demyelination was particularly pronounced near the pial surface and the midline between the hemispheres. In EAE + GA mice, myelin was typically intact with only minor demyelination, MBP-median fluorescence density 26% lower than in naïve mice. Kruskal-Wallis one-way ANOVA of MBP fluorescence density indicated a significant difference between the groups [χ^2^_(2)_ = 26.602; p < 0.001]; Dunn’s pairwise (Bonferroni corrected) post hoc comparison indicated significantly less MBP expression in untreated EAE-mice than in both naïve (p < 0.001) and GA-treated mice (p < 0.001); naïve and GA treated mice did not differ (p = 1.000).

To detect neuronal death, we stained for receptor interacting protein kinase 3 (RIP3), signaling apoptosis and necroptosis pathways^34^, and assessed its expression in distinct segments of the hippocampus. Robust RIP3 expression was evident in part of the hippocampal neurons (NeuN^+^) in EAE-untreated mice, exposing the neuronal injury inflicted by the disease, and it was less pronounced in EAE-mice treated with GA (depicted in the CA1 and CA3 in Fig. 7A,B). Quantification of necroptotic neurons was performed by counting the number of RIP3 positive cells from the total number of neurons (NeuN positive) in CA1 (47,850 μm^2^ per section), CA2 (12,960 μm^2^ per section), CA3 (59,400 μm^2^ per section), and CA4 (11,250 μm^2^ per section), 6–8 quantifications from both hemispheres per mouse, 3–7 mice per group (Fig. 7C).

To assess the effects of EAE induction and GA-treatment on neuronal death across the overall hippocampal formation, a two-way ANOVA was performed for treatment groups (between subject factor: naïve/EAE-untreated/EAE + GA), sub-regions (within subjects factor: CA1/CA2/CA3/CA4) and their interaction (Treatment × Sub-regions) on RIP3 levels. These analyses revealed a significant main effect only for treatment [F_(2,8)_ = 7.541; p = 0.014]. Between subjects post-hoc Scheffe comparisons indicated that the hippocampus overall RIP3 levels of EAE-untreated mice (regardless of sub-regions) was significantly higher than that of naïve (p = 0.015) but did differ from GA-treated (p = 0.140) mice. Hippocampal overall RIP3 levels did not differ between naïve and EAE + GA-treated mice (p = 0.461). One way ANOVA analyses sought for differences in necrotopic neurons between the groups revealed significant differences in the CA1 [F_(2,12)_ = 9.899; p = 0.003], CA2 [F_(2,12)_ = 14.763; p = 0.001] and CA3 [F_(2,10)_ = 10.903; p = 0.005], but non-significant differences in the CA4 [F_(2,13)_ = 1.068; p = 0.377]. Scheffe post-hoc comparisons indicated significant increase in EAE-mice compared to naïve, in the CA1 (p = 0.010), CA2 (p = 0.001), and CA3 (p = 0.006). A significant decrease from EAE following GA-treatment was obtained in the CA1 (p = 0.018) and CA3 (p = 0.040). A significant difference between naive and GA-treated mice RIP3 levels was evident only in CA2 (p = 0.050); these groups’ RIP3 levels did not differ in the CA1 (p = 1.00) and CA3 (p = 0.603).

Collectively, these immunohistochemical analyses indicate that the disease pathological characteristics, inflammation, demyelination and neuronal death, are evident in the frontal cortex and the hippocampus 21 days following EAE induction, in a manner that coincides with the poor performance in the T-maze task, supporting a linkage between cognitive impairments and gray matter tissue injury in this model.

## Discussion

Cognitive impairments are currently recognized as key symptoms of MS^3–7^, but systematic analyses of specific cognitive deficits, their connection to tissue injury, and the ability to ameliorate them by therapy, are lacking. Animal models could be useful in this regard, however, the severity of the motor impairments in the EAE animal model of MS impedes its utilization for such studies. In the current study, we devised a behavioral system that evaluates cognitive functions in EAE-affected mice irrespective of their motor performance, enabling specific assessment of cognitive deficits along the disease duration, their associated brain damage, and the consequences of an immunomodulatory treatment on these particular manifestations.

The DNMS T-maze task and the experimental design were adjusted to minimize the physical demands, focusing on the choices the mice made, without considering indices affected by their motor dysfunction. In each ‘choice’ run, the mice had to recall where they were forced to turn in the previous ‘forced’ run relying on their working memory. In order to remember the DMNS rule over the daily sessions, they had to rely on their long term memory function. Accordingly, this approach exposed working and long term memory deficits in EAE-affected mice, irrespective of their motor impairments. The findings, collected along the disease course, indicate that EAE infliction, as such, leads to substantial cognitive impairments, manifested by a drastic decline in task performance, approaching the 50% chance and the performance exhibited at the early learning sessions, thus “erasing” the learning phase. This decline is not related to the time that elapsed from training or the intervals between testing sessions, since the EAE-mice performed significantly worse than naive mice which underwent testing at the same intervals.

A significant decline in T-maze task performance was evident only at the “clinical disease phase”, whereas seven days after disease induction, before motor symptoms onset, the reduction in the number of correct responses was not statistically significant compared to the performance of the same mice at the last training session as well as of naïve mice. Previous studies that tested memory functions in EAE-induced mice using the Morris water maze reported impairments at the pre-clinical stage^14,35^. This discrepancy may result from differences between these tasks in terms of stress, motor and cognitive demands. Our findings support the notion that cognitive impairment does not precede the disease onset.

Following the appearance of clinical symptoms, 14 days after EAE induction, task performance drastically declined among EAE-untreated mice, and this impairment was sustained along the disease course. Other studies that used the Barnes Maze^15^ and the Morris water maze^36^ described impairments in learning and memory only at later disease stages (40 and 55 days from induction). This discrepancy may result from differences in the cognitive demands those tasks require. Alternatively, the discrepancies may result from the low disease burden of the mice which could be tested using those physically demanding systems (clinical scores less than 2 and less than 1, in those studies respectively). In contrast, our behavioral setup, which enabled assessment of mice even with EAE grades of 3–3.5 (hind leg paralysis with/without hind body paresis), irrespective of their motor functions, facilitated the exposure of cognitive deficits already at the early disease phase. Our findings are in accordance with findings in MS patients, which detected cognitive deficits already at the earliest clinical stages^37,38^. Notably, with EAE aggravation, the impairment increased, as revealed by the significant linear correlation between the overall T-maze performance and the overall motor impairments, indicating that the loss of cognitive functions is strongly associated with disease severity. In MS as well, patients with severe disease generally display more pronounced and pervasive cognitive deficits^5,6,9^.

The cognitive impairments found in EAE-affected mice are least likely to result from anxiety, which is known to alter cognitive functions^28,29^, since assessments of anxiety-like behaviors using the open-field test, a day after the T-maze task performance had declined (day 15), indicated that EAE-affected mice did not differ from naïve mice in the time spent or the relative distance moved in the center. In addition, in a preliminary experiment, task performance of mice injected only with Freund’s adjuvant enriched with Mycobacterium Tuberculosis was similar to that of naïve mice (not shown), indicating that sickness behavior and pains which could also affect cognitive performance did not affect the results. Our combined findings thus indicate that in addition to the motor impairment inflicted by EAE, the disease process leads to substantial working and long-term memory impairments, starting from the early stages and increasing with disease progress, similarly to that observed in MS patients.

Given the recognized importance of cognitive dysfunction in MS pathology and the notion that disease-modifying therapies should be also evaluated for their ability to target cognitive aspects, we tested the consequences of GA-treatment on this particular manifestation. Our findings indicate that GA conserved the cognitive functions, so that the treated-mice performed significantly better than EAE-untreated mice. It should be noted that a slight (not significant) gradual reduction in task performance was noticed with time in the GA-treated mice, at days 21 and 28. This could result from genuine memory impairment exhibited later due to the preventive effect of GA, or from the extended time duration between the consecutive learning and testing sessions. Indeed, to a certain extent, parallel reduction was observed also in naïve mice. In any case, throughout the experiment course, task performance of the GA-treated mice did not change significantly, and did not differ from that of that of the naïve controls.

It should be noted that in the current experiment, GA-treatment did not entirely prevent EAE manifestations, so the treated-mice exhibited explicit motor dysfunction, reaching a clinical score of nearly 2, although significantly lower than that of the EAE-untreated mice. Nevertheless, the difference in task performance between these two groups is less likely to be an outcome of the difference in clinical scores. This is indicated by the lack of a significant linear regression between the overall task performance and the overall motor impairment in the GA-treated mice, in contrast to the significant regression obtained for the EAE-untreated mice. Thus, whereas in EAE-untreated mice, working memory performance is strongly associated with EAE severity, in the GA-treated mice this cognitive function is unrelated to the disease burden. Moreover, when the overall T-maze task performances of mice presenting similar motor impairments were compared, only the EAE-untreated mice exhibited a significat reduction comapred with naïve mice (p < 0.001), whereas the GA-treated mice did not differ from the naïve controls. These combined analyses imply that in terms of the cognition functions assessed in this study, the EAE-untreated and EAE + GA-treated mice are two distinct populations. Accordingly, in addition to the ameliorating effect of GA on motor impairments, this treatment conserves cognitive capacities and prevents its disease-related typical deterioration.

Our findings are in accord with previous studies in experimental animal models reporting that GA-treatment prevented short term memory decline in EAE-mice with low clinical scores^16^, and ameliorated the cognitive deficits induced by chronic cerebral hypoperfusion^39^ and by cranial-irradiation, in rats^40^. In a mouse model of Alzheimer’s disease, GA-treatment reduced the cognitive decline assessed by Morris water maze and plaque formation^41^. MS patients treated with GA have shown stable cognitive performance during 10 years of evaluation^42^. An additional study reported benefits of GA-treatment in regards to cognitive improvements^43^. However, long term placebo controlled conclusive studies are still required to establish such effect in MS patients. It should be noted that in the present study treatment was initiated before the appearance of clinical manifestations, although after disease induction. Our findings indicating that cognitive impairment does not precede the disease onset, as well as the findings in MS patients, where prominent cognitive deficits are generally displayed in the advanced disease stages^5,6,9^ support the notion that early treatment may be effective in arresting cognitive deterioration.

Inflammation is widely implicated as the primary mechanism mediating MS/EAE^44,45^. In the current study, immunohistochemical analyses exposed the hallmark of CNS inflammation, T-cell infiltration and extensive gliosis, in EAE-mice, in the frontal cortex and the hippocampus, brain regions whose integrity was associated with correct performance of the DNMS T-maze task^30–33^. Moreover, these indices of CNS inflammation were down-regulated in GA-treated mice. These findings support a role for focal inflammation in the cognitive impairments detected using the T-maze task, in accord with a recent study that used MRI parameters and showed a connection between focal inflammation and cognitive loss in MS patients^46^. One of the ways by which focal inflammation contributes to cognitive impairment has been attributed to the lack of fine-tuning between the blood supply and the neuronal needs, elucidated in our recent studies^27^. We found that in the cortex of EAE-affected mice, perivascular astrocytes, activated by pro-inflammatory mediators such as GMCSF, detach from the blood vessels and lose their structural confinement to the functional neuronal boundaries, thus failing their essential role in neuro-hemodynamic coupling. This obstructed crosstalk between the blood vessels and the neurons may contribute to the cognitive decline occurring in EAE/MS.

In addition to the typical inflammation, in EAE-untreated mice, genuine gray matter tissue injury was manifested by cortical demyelination and hippocampal neuronal death in the CA1-3 sub-regions of the hippocampus (evident by robust expression of RIP3 signaling apoptosis and necroptosis pathways)^34^. The involvement of CA1, and CA3 sub-regions of the hippocampus in memory functions has been demonstrated^47,48^. Axonal damages evident by amyloid precursor protein (APP) expression had been previously shown in this model^49^, suggesting that altered functionality could also contribute to the neurodegeration in the EAE-induced mice. Neurodegeneration is considered the major determinant of cognitive dysfunction in MS patients^50^. Both types of tissue damages, demyelination and degeneration, were significantly ameliorated by GA-treatment. Thus, the disease pathological characteristics - inflammation, demyelination and neurodegeneration - which are evident in the frontal cortex and the hippocampus of EAE-mice, are downregulated by treatment, in a manner that coincides with improved T-maze task performance.

It should be noted that the prevalence of cell infiltration and demyelination in the cortex of C57BL/6 mice induced by the MOG peptide-EAE model is debatable^45^. However, our previous findings utilizing immunohistochemistry and MRI^27,49^, as well as the immunohistochemical analysis preformed in the current study, revealed immune cell infiltration (penetrating from the pia mater, or through blood capillaries extending from white matter regions), along with demyelination (mainly in the layers adjacent to the pial surface and to the midline between the hemispheres), in the frontal cortex of these mice.

The way by which GA affects cognitive functions can be attributed to its immunomodulatory properties and its ability to down-regulate the detrimental inflammation^17^. GA-treatment was shown to restore the impaired vascular-neuronal connections and the structural confinement of the astrocytic processes to the functional borders^27^, so it may also affect cognition by reestablishing the neuro-hemodynamic coupling. Furthermore, accumulated findings indicate that GA-treatment generates in the CNS neuroprotective consequences, such as reduced neuronal damages and proliferation of neuronal progenitor cells (neurogenesis) that migrate to injury sites^24^. GA-treatment induces also elevation of brain derived neurotrophic factor (BDNF)^19,20^, which was shown to modulate the synaptic function and plasticity critical for cognition and memory^51^. These neuroprotective effects may counteract the neurodegenerative disease course that underlies the cognitive deterioration.

A significant aspect of this study is the ability to assess, for the first time, cognitive deficits in EAE-affected mice throughout the disease duration, irrespective of the predominant motor dysfunction. This enabled us to establish the linkage between the cognitive impairments and the gray matter tissue injury occurring in this disease as well as the ability to affect them by treatment. This system can be further utilized to investigate cognitive functions in various MS models, such as relapsing remitting EAE, and the linkage between the disease form/phase, cognitive deficit and CNS tissue damage.

## Materials and Methods

### Mice

C57BL/6 mice were purchased from Envigo (Jerusalem, Israel) and maintained under specific pathogen free (SPF) housing conditions, on a reverse 12h-12h light-dark cycle. Food and water were given *ad libitum* (unless specified otherwise). Training and testing began at nine weeks of age, after acclimation to the housing conditions. Behavioral assessments were performed in designated experimental rooms away from the *vivarium*, during the dark phase, following one hour of habituation to the test room. All experiments were approved by the Institutional Animal Care and Use Committee of the Weizmann Institute and performed in accordance with the relevant guidelines and regulations.

### EAE induction and evaluation

EAE was induced by the peptide encompassing amino acids 35–55 of myelin oligodendrocyte glycoprotein (MOG), synthesized by Genscript (Piscataway, NJ). Mice were injected subcutaneously with 100 µl emulsion containing 200 µg of the peptide in Freund’s adjuvant enriched with 3.3 mg/ml heat-inactivated Mycobacterium Tuberculosis (Sigma, St. Louis, MO). Pertussis toxin (Sigma), 150 ng/mouse, was injected intraperitoneally immediately after the encephalitogenic injection and 48 h later. Mice were examined daily, and EAE was scored as follows: 1 - loss of tail tonicity, 2 - hind limb weakness or partial paralysis, 3 - hind leg paralysis, 3.5 - hind leg complete paralysis with hind body paresis, 4 - hind and foreleg paralysis, 5 - death.

### Glatiramer acetate (GA)

GA containing four amino acids L-alanine, L-glutamate, L-lysine, and L-tyrosine was obtained from Teva Pharmaceutical Industries (Petah Tiqva, Israel). GA-treatment, 2 mg/mouse (in phosphate buffered saline, PBS), was injected subcutaneously for seven days, beginning at the day of disease induction. Mice not treated by GA were similarly injected by PBS alone. An outline of disease induction and treatments is shown in Fig. 1A.

### T-maze

The delayed-non-matching to sample (DNMS) T-maze task was adapted from Deacon and Rawlins^52^. A “T” shaped maze apparatus consisting of a stem start arm and two branching side arms (10 W, 45 L, 10H cm, each), equipped with three sliding doors was used (Fig. 1B). Mice were habituated to the apparatus for three days (15 min/day). To increase the animals’ motivation, food was deprived over the light phase that preceded the training/testing sessions (food restriction on alternating days, food supply restored immediately following the training/testing).

Mice were trained to locate and collect a reward that was hidden at the end of one of the side arms. The reward was a crumb of a “Western diet” food pellet (cat # D12451, Research Diets, New Brunswick, NJ), to which they were previously introduced in the home cage. A training session was comprised of six trials, each consisted of two runs: a ‘forced’ run in which only one arm was accessible, so the mice were forced to turn to collect the reward (e.g. left panel in Fig. 1B), and a ‘choice’ run that followed immediately in which both arms were accessible and the mice had to choose which arm to go into to obtain the reward (e.g. the right panel in Fig. 1B). Whenever a mouse fully crossed the threshold of an arm (all four paws over the ‘line’), a sliding door was shut behind, making this choice ‘final’. In the DMNS task ‘choice’ runs, the reward was always located at the opposite arm from which it was placed in the previous ‘forced’ run (left or right, varying randomly). Thus, the mice had to learn that if in a ‘forced’ run they were rewarded on the left side, they had to turn right in the next ‘choice’ run in order to collect the reward (and *vice versa*). The mice learned the DNMS T-maze task in seven training sessions (L1-L7), every other day. The inclusion criterion to take part in the later testing sessions was at least five correct responses out of six choice runs for both L6 and L7.

Mice performance in the DNMS T-maze was evaluated four times (T1–T4; Fig. 1A) on days 7, 14, 21, and 28 post EAE induction. Each test session consisted of six trials (‘forced’ run followed by a ‘choice’ run). The number of correct choices was the mice cognitive functions index. To test cognitive functions in EAE-affected mice, when the motor impairment impedes mobility, we adjusted the T-maze apparatus by relocating the start and reward positions closer to the T junction (10 cm instead of 45 cm, Fig. 1B). In addition, for mice with clinical grades of two and above, no time limitation to complete a run was imposed.

### Anxiety tests

Anxiety-like behaviors were assessed in parallel to the DNMS T-maze assessments, i.e. one day after the end of the learning phase (day −1) using the elevated-plus maze (EPM), one day after T1 (day 8 from EAE induction) using the dark/light transfer test (DLT), and one day after T2 (day 15 from EAE induction) using the open-field test (OF). The mice exploratory behavior was monitored and scored automatically, using a video tracking system (VideoMot2, TSE Systems). Protocols were adapted from Elliot and colleagues^53^.

The EPM apparatus, a gray polyvinyl chloride (PVC) maze, comprised of a central part (5 × 5 cm), two opposing open arms (30.5 × 5 cm) and two opposing closed arms (30.5 × 5 × 15 cm). Mice were placed in the center, facing an open arm to initiate a 5 min test session under dim illumination (6 lux). The more a mouse explored the maze and/or the more time it spent in the open arms and/or the more frequently it visited the open arms, the less anxious it was considered to be.

The DLT test apparatus, a PVC box, was divided into a black dark compartment (14 × 27 × 26 cm) connected to a white 700 lux illuminated lit compartment (30 × 27 × 26 cm). Mice were placed in the dark compartment to initiate a 5 min test session. The more a mouse explored the lit section and/or the more time it spent in the lit section and/or the more frequently it visited the lit section, the less anxious it was considered to be.

The OF arena, a white PVC box (50 × 50 × 22 cm), was lit to 120 lux. The mice were placed in the box corner to initiate a 10 min test session. The more a mouse explored the arena and/or the more time it spent in the center of the arena and/or the more frequently it visited the center of the arena, the less anxious it was considered to be.

### Immunohistochemistry

Animals were deeply anesthetized. Brains were dissected, postfixed in paraformaldehyde (2.5% for 48 hr, and then 1% for one week, at 4 °C), paraffin embedded and sectioned coronally (4 μm) by a microtome (Leica, Nussloch, DE). Paraffin sections were deparaffinized and rehydrated. Antigen retrieval was performed in 10 mM citric acid pH 6 or Tris EDTA pH 9, for 10 min in a microwave, to break protein crosslinks and to unmask the antigens. After pre-incubation with 20% normal horse serum and 0.2% Triton X-100, sections were incubated with the primary antibodies at RT for 24 hr, followed by an additional incubation at 4 °C for 48 hr. The following primary antibodies were used: rabbit anti-glial fibrillary acidic protein (GFAP, 1:100, Dako, Glostrup, Denmark), mouse anti-NeuN (1:25, Millipore, Temecula, CA), rabbit anti-Iba1 (1:100, Wako, Richmond, VA), rat anti MBP (1;50, abcam, Cambridge, UK), rabbit anti-CD3 (1:50, abcam), rabbit anti-receptor interacting protein kinase 3 (RIP3, 1:200, abcam). The second antibody step was performed by labeling with species specific highly cross-absorbed cy2 or cy3 conjugated antibodies (1:100, Jackson ImmunoResearch, West Grove, PA) for 30–60 min. In some cases, the signal was enhanced by incubation with biotinylated secondary antibodies for 90 min, followed by cy2 or cy3 conjugated streptavidin (1:100, Jackson ImmunoResearch). Sections were counterstained with Hoechst 33258 (Molecular Probes, Eugene, OR) for nuclear labeling. Stained sections were examined and photographed with a fluorescence microscope (Eclipse Ni-U, Nikon, Tokyo, Japan) equipped with Plan Fluor objectives, connected to a monochrome camera (DS-Qi1, Nikon).

Fluorescence density of MBP expression was measured in prefrontal cortex sections stained with anti-rat MBP (in areas of 27,900 μm^2^, 6–8 measurements from both hemispheres per mouse, four mice per group), using Image Pro Plus 4.5 software (Media Cybernetics, Bethesda, MD). The percentage of RIP3 expressing cells was quantified by counting the RIP3 expressing cells from the number of NeuN expressing cells in the CA1, CA2, CA3, CA4 subfields of the hippocampus in an area of 47,850 μm^2^, 12,960 μm^2^, 59,400 μm^2^, 11,250 μm^2^, respectively (6–8 quantifications from both hemispheres per mouse, 3–7 mice per group).

### Statistical analyses

The data were analyzed by SPSS software (version 23, IBM, Armonk, NY), Statistica (version 12, Statsoft). The data were first tested for normality using the Shapiro-Wilks test. If the data were normally distributed, parametric comparisons were performed: independent samples t-test and one-way or two-way analysis of variance (ANOVA) followed by Scheffe post-hoc comparisons. When the data deviated significantly from normality, non-parametric tests were applied: Kruskal-Wallis one-way ANOVA followed by Dunn’s pairwise comparison post hoc analysis or Friedman’s two-way ANOVA by ranks followed by Wilcoxon signed-rank tests pairwise comparisons. Data distribution is presented in box plots. The center lines in the boxes show the medians, box limits indicate the 25^th^ and 75^th^ percentiles, whiskers indicate the minimal and maximal values. Data presented as the mean is depicted with the standard error of the mean (SEM). A probability value (p) of less than 0.05 was considered significant.

## Supplementary information

Supplemental
