# Supplementary material for: Cognitive impairment in an animal model of multiple sclerosis and its amelioration by glatiramer acetate
Source: Sci Rep. 2019 Mar 11;9:4140. doi: 10.1038/s41598-019-40713-4 (PMC6412002; doi:10.1038/s41598-019-40713-4)
Supplement: Supplementary file 1 — Supplemental [file 41598_2019_40713_MOESM1_ESM.pdf]

Supplementary information

Cognitive impairment in an animal model of multiple sclerosis and its amelioration by glatiramer acetate

Rina Aharoni, Nofar Schottlender, Dekel D. Bar Lev, Raya Eilam, Michael Sela, Michael Tsoory, Ruth Arnon

The effect of EAE induction and GA treatment on anxiety-like behaviors. Anxiety-like behaviors were assessed at several time points, in parallel to the DNMS T-maze assessments i.e. at the end of the learning phase (one day before disease induction, anx1) using the elevated-plus maze (EPM), one day after T1 (day 8 from EAE induction, anx2) using the dark/light transfer test (DLT), and one day after T2 (day 15 from EAE induction anx3) using the open-field test (OF).

At the end of the learning phase (L7), before disease induction, the elevated-plus maze indices exhibited by all three groups were similar (Fig. 6A). One way ANOVA indicated that the groups did not differ in any of the monitored indices, namely, the time spent in the open arms [$F_{(2,6)}$=1.012; p=0.418], the relative distance moved in the open arms [$F_{(2,6)}$=1.034; p=0.411], and the total distance moved [$F_{(2,6)}$=1.753; p=0.251], thus corroborating equal allocation of mice into experimental groups in terms of anxiety, motor function and the above noted DNMS task performance.

Eight days after disease induction, before clinical symptoms manifestation, dark/light transfer test revealed that mice which had undergone the EAE induction process (either untreated or GA-treated) exhibited anxiety-like behavior (Fig 6B). This was indicated by a reduction in the time spent as well as in the distance moved in the lit area, but not in the time spent per visit in the lit area. Kruskal–Wallis and ANOVA analyses indicated that the EAE-untreated and EAE+GA groups did not differ from each other, yet differed from the naive group in the time spent in the light [$\chi^2_{(2)}$=9.979;p=0.007; mean ranks- naive=14.00, EAE=5.20, GA=6.67; Dunn's (Bonferroni corrected) pairwise comparisons: naive>EAE-untreated (p=0.010), naive>EAE+GA (p=0.030), EAE-untreated ~ EAE+GA (p=1.00)] and distance moved in light [$F_{(2,13)}$=27.904; p<0.001; Scheffe post-hoc comparisons: naive>EAE-untreated (p<0.001), naive>EAE+GA (p<0.001), EAE-untreated ~ EAE+GA (p=0.766)]; yet all groups did not differ in the time spent per visit in the lit section [$F_{(2,13)}$=1.067; p=0.372].

Fifteen days after disease induction, although clinical manifestations were clearly apparent, the anxiety parameter obtained in the open-field test, namely, time in the center as well as the relative distance in the center (from the total distance traveled), for all three groups were similar (Fig. 6C). It should be noted that the total distance moved by the two EAE-induced groups (both the untreated and the GA-treated) were considerably lower than that of the naïve mice, corroborating their motor dysfunction at this time point. One way ANOVA indicated that all three groups (naïve, EAE-untreated, and EAE+GA) did not differ in time spent in the center [$F_{(2,13)}$=0.927; p=0.421] or in relative distance moved in the open-field center [$F_{(2,13)}$=0.967; p=0.406]. Similarly, it indicated that the untreated and GA-treated groups did not differ from each other, yet differed from the naive group in the total distance moved [$F_{(2,15)}$=62.010; p<0.001; Scheffe post-hoc comparisons: EAE-untreated ~ EAE+GA (p<0.001), naive>EAE-untreated (p<0.001), naive>EAE+GA (p<0.001)].

Collectively, the anxiety indices data indicate that the differences in the T-maze task performance found between EAE-affected mice, untreated as opposed to GA treated, are unlikely related to differences in anxiety.

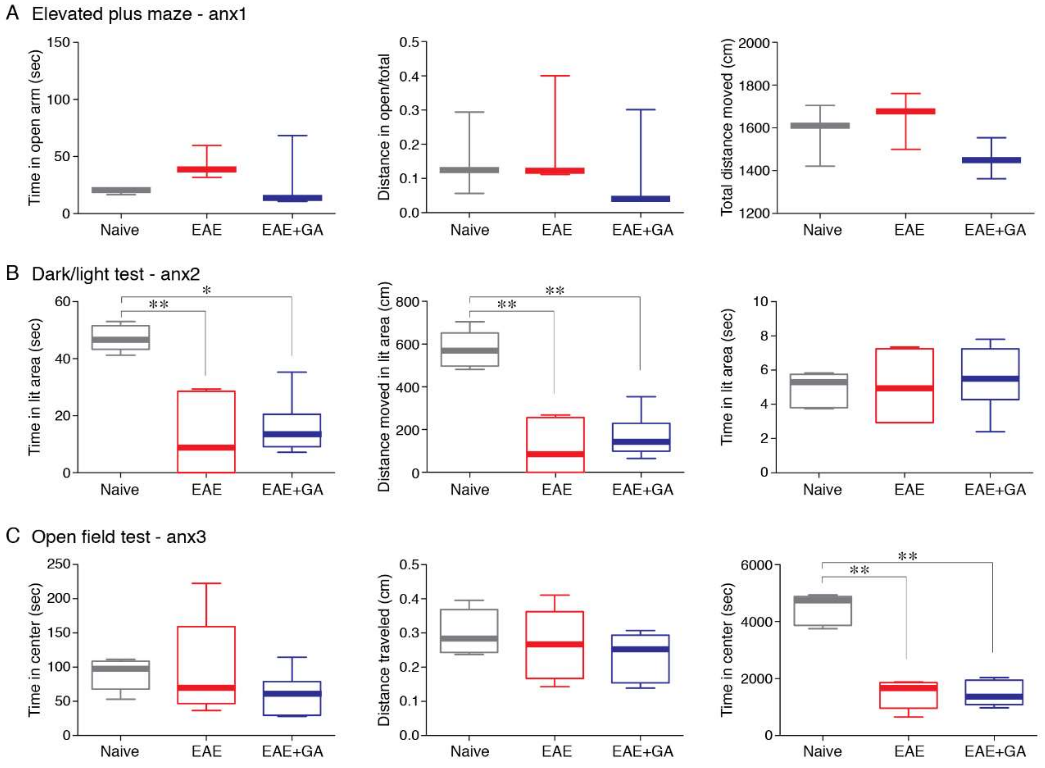

Supplementary Figure 1. The effect of EAE induction and GA treatment on anxiety-like behaviors. (A) Elevated-plus maze (EPM) - anx1, applied at the end of the learning phase (one day before disease induction). (B) Dark/light transfer test (DLT) - anx2, applied 8 days after EAE induction (one day after T1), (C) open-field test (OF) - anx3, applied 15 days after EAE induction (one day after T2). 3-6 mice per group in each test. *indicates significant differences (* $p<0.05$, ** $p<0.01$).
